# Drug Development for Alzheimer’s and Parkinson’s Disease: Where Do We Go Now?

**DOI:** 10.3390/pharmaceutics16060708

**Published:** 2024-05-24

**Authors:** Lisa Sequeira, Sofia Benfeito, Carlos Fernandes, Inês Lima, Joana Peixoto, Catarina Alves, Cláudia Sofia Machado, Alexandra Gaspar, Fernanda Borges, Daniel Chavarria

**Affiliations:** CIQUP-IMS—Centro de Investigação em Química da Universidade do Porto, Institute of Molecular Sciences, Department of Chemistry and Biochemistry, Faculty of Sciences, University of Porto, R. Campo Alegre s/n, 4169-007 Porto, Portugal

**Keywords:** neurodegenerative diseases, Parkinson’s disease, Alzheimer’s disease, drug discovery, disease-modifying drugs

## Abstract

Neurodegenerative diseases (NDs) are a set of progressive, chronic, and incurable diseases characterized by the gradual loss of neurons, culminating in the decline of cognitive and/or motor functions. Alzheimer’s disease (AD) and Parkinson’s disease (PD) are the most common NDs and represent an enormous burden both in terms of human suffering and economic cost. The available therapies for AD and PD only provide symptomatic and palliative relief for a limited period and are unable to modify the diseases’ progression. Over the last decades, research efforts have been focused on developing new pharmacological treatments for these NDs. However, to date, no breakthrough treatment has been discovered. Hence, the development of disease-modifying drugs able to halt or reverse the progression of NDs remains an unmet clinical need. This review summarizes the major hallmarks of AD and PD and the drugs available for pharmacological treatment. It also sheds light on potential directions that can be pursued to develop new, disease-modifying drugs to treat AD and PD, describing as representative examples some advances in the development of drug candidates targeting oxidative stress and adenosine A2A receptors.

## 1. Introduction

Neurodegenerative diseases (NDs) are a set of progressive, chronic, and incurable neurological disorders characterized by the loss of neurons and synaptic connections, which irreversibly produce a series of events commonly related to motor disability, cognitive impairment, and dementia [1]. They represent an enormous disease burden, both in terms of human suffering and economic costs [2], being the foremost contributors to incapacity and dependence due to their debilitating nature [3]. The most common NDs include Alzheimer’s disease (AD), Parkinson’s disease (PD), Huntington’s disease (HD), and Amyotrophic Lateral Sclerosis (ALS). 

**Figure 1:**
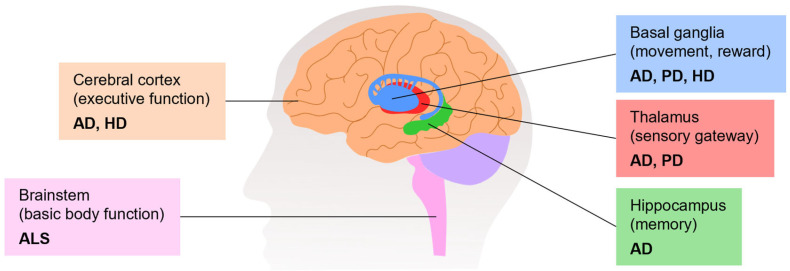
Primary brain regions affected in the most common neurodegenerative diseases. Adapted from [6].

The etiology of NDs is not completely understood and the onset of neurodegeneration may precede the clinical symptoms by many years. However, it is generally accepted that the pathogenesis of NDs is multifactorial, involving a complex combination of genetic, environmental, and endogenous factors acting cooperatively or independently [4,5]. Although each disease presents its particular molecular mechanisms and clinical manifestations (Figure 1), NDs share common pathogenic events [6].

Despite the intensive research performed so far, to date, no breakthrough treatment has been discovered. The available therapies for NDs only provide symptomatic and palliative relief for a limited period [7] and are unable to modify the disease progression [8,9]. Therefore, the development of disease-modifying drugs able to prevent, halt or reverse the progression of NDs remains an unmet clinical need. In this review, we summarize the major hallmarks of AD and PD, the drugs available for pharmacological treatment, and future directions for the development of new and disease-modifying drugs to treat these NDs. 

## 2. Alzheimer’s and Parkinson’s Diseases: The Epidemiologic Forecast

The average human lifespan of the worldwide population has been rising in recent decades [10]. Several factors seem to contribute to this success, namely the cumulative progress in sanitation and medical care, rising living standards, and the decline in child mortality [10,11]. Although the increased life expectancy must be celebrated, a proportional rise in the frequency and prevalence of NDs is expected [10,12]. Therefore, the rise of elderly populations has been soaring with the increased incidence of age-related degenerative diseases, reaching epidemic proportions in high-income countries [13]. Several genetic risk factors, lifestyles, and environmental exposure to a diversity of pollutants are implicated in the neurodegeneration process [14]. In 2020, the worldwide population with age higher than 65 years was estimated to be 727 million, a 195 million increase since 2010. Over the next three decades, the number of worldwide elderly is projected to more than double, reaching over 1.5 billion in 2050. By mid-century, one in six people globally will be aged 65 years or older [15]. Although the increase in longevity represents a progress *per se*, it can become a social, economic, and medical burden when it is not associated with the maintenance of the quality of life. The World Health Organization indicated that central nervous system (CNS) diseases are the major medical challenge of the 21st Century. Among them, NDs, namely AD and PD, are the most prevalent CNS disorders [16]. 

The World Health Organization recognized AD as the most common form of dementia in the elderly, accounting for 50–56%, and a major cause of death, being considered one of the greatest global public health challenges [17,18]. Currently, the number of people aged 65 and older affected by AD dementia is more than 55 million worldwide and it is expected that this number will rapidly increase to 132 million by 2050 [19,20].

The overall number of people diagnosed with PD has also been growing progressively at a global level. In 2019, approximately 8.5 million individuals received a PD diagnosis. Estimates suggest that, in 2019, PD resulted in 5.8 million disability-adjusted life years, an increase of 81% since 2000, and caused 329,000 deaths, an increase of over 100% since 2000. This estimation is expected to increase to 12 million people in 2050 [21,22].

## 3. Major Hallmarks of Alzheimer’s and Parkinson’s Diseases

### 3.1. Alzheimer’s Disease

Alzheimer’s disease was first diagnosed in 1906 by Dr. Alois Alzheimer, when he noticed changes in the brain tissue of a woman who had died of an unusual mental illness [23]. 

AD is an irreversible, complex, and progressive ND that results in cognitive impairment and memory injury. Despite its prevalence among the elderly, AD dementia is distinct from a normal aging process [24]. The progression of AD can be divided into three stages. The first is often mistakenly attributed to age-related upsets or manifestations of stress [25]. In this stage, the patient has memory lapses such as forgetting familiar words or the location of everyday objects, which denotes its lack of ability to produce new memories and skills [26,27]. The second stage of AD is typically the longest one and can last for many years. Herein, the progressive deterioration of neurons can lead to problems with speech and severe difficulties in reading and writing. During this phase, memory problems worsen, and the patient may fail to recognize close relatives [28]. In the most advanced phase, AD patients show loss of cognitive and motor functions, confusion, and disorientation, with most patients having mobility problems, hallucinations, and delirium, leading to absolute dependence on 24 h supervision, hospital care, and unavoidable death [29,30]. 

Although the specific cause of AD is still unknown, it is well recognized that a multiplicity of pathological stimuli can play a key role, which causes an increased risk of disease development [31,32]. Age and family history of the disease are considered the strongest risk factors for familial and sporadic AD [33]. The presence of the ε4 allele of the apolipoprotein E4 (ApoE4) genotype, found on chromosome 19, appears to be a primarily risk factor for patients with sporadic AD [34,35,36]. In addition, genetic mutations in APP on chromosome 21, presenilin-1 (PSEN-1) on chromosome 14, and presenilin-2 (PSEN-2) on chromosome 1 can also cause familial AD [37,38]. Other putative risk factors include head trauma, depression, diabetes mellitus, hypothyroidism, and a series of vascular factors [39,40].

A conclusive diagnosis of AD requires a detailed post-mortem microscopic examination of the brain [37]. However, AD can be currently diagnosed with more than 95% accuracy in living patients by carefully analyzing the patients’ family history, assessing cognitive function with neuropsychological tests, and evaluating AD biomarkers, namely with high-tech neuroimaging data or cerebrospinal fluid analysis [41,42]. 

**Figure 2:**
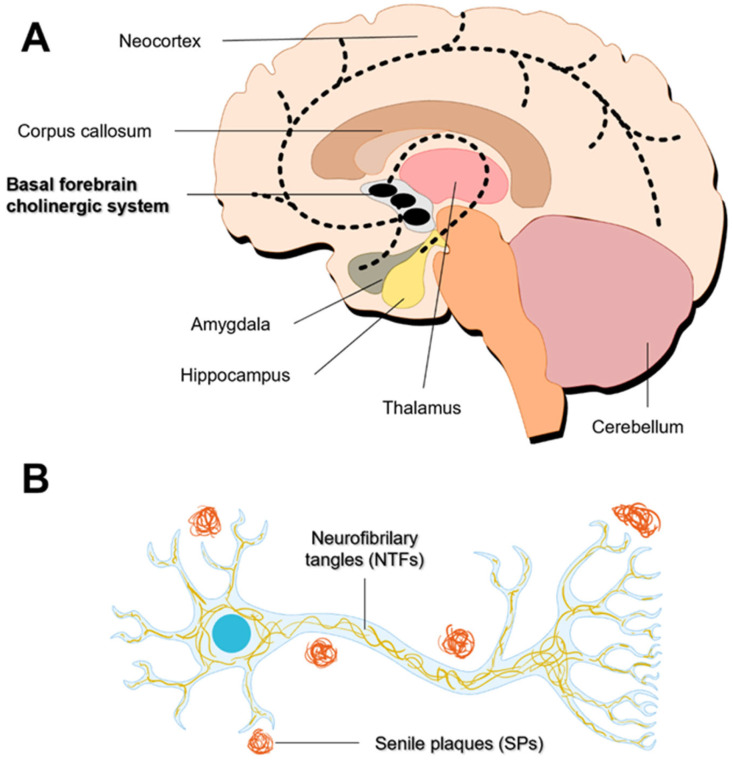
Major hallmarks of AD. (**A**) Degeneration of cholinergic neurons in the basal forebrain. (**B**) Formation of senile plaques and neurofibrillary tangles. Adapted from [52].

The progressive cognitive impairment observed in AD patients can be associated with the significant reduction of brain size [43]. The brain atrophy arises from the loss of synapses and from the selective neuronal death in the hippocampus and in the cerebral cortex [43,44,45,46]. The most prominent losses are observed in neurons with long projections, such as cholinergic neurons in the basal forebrain (Figure 2A) [47]. These neurons innervate the hippocampus, thalamus, amygdala, and neocortex, and play key roles in attention, cognitive flexibility, and learning [48]. Although the neurons that degenerate in AD are mostly cholinergic [49], glutamatergic neurons are also affected [50].

AD is characterized by extensive atrophy of the brain caused by two main neuropathologic changes—the formation of amyloid plaques (also called senile plaques) and the appearance of neurofibrillary tangles (NFTs)—that lead to neuronal loss and synaptic changes in brain-specific areas essential for cognitive and memory functions (Figure 2B) [51]. 

The amyloid plaques result from the abnormal extracellular accumulation and deposition of insoluble aggregates of fibrillar β-amyloid (Aβ) [53]. Sequential cleavage of the amyloid precursor protein (APP) in the cell membrane by the enzymes-β and Ƴ-secretases gives rise to the family of Aβ peptides (most commonly, 40–42 amino acids in length) [54,55]. Although the formation of Aβ is believed to be a physiological process in normal aging [56], Aβ1-42 isoform was identified as a major contributor to the disease process [57,58]. 

Intracellular NFTs are formed by aggregated misfolded tau protein (tau-P), the major microtubule-associated protein predominantly found in the axons of mature neurons [59]. In AD, tau hyperphosphorylation induces a loss of function that hampers its ability to bind to microtubules, leading to microtubule depolymerization that compromises the axonal trafficking and the dendrite structure [60]. When tangle-bearing neurons die, NFTs become extraneuronal and activate a series of neurotoxic processes that can cause synaptic dysfunction and neuronal death [61]. Overall, AD brains show a decline in neuronal mass in regions related to cognition and memory, which leads to a depletion of cholinergic neurons and acetylcholine (ACh), resulting in synaptic dysfunction [47,62]. 

Other pathological features also play a crucial role in the progress of AD, including cholinergic deficit, enhanced brain oxidative stress, the overproduction of free radical, mitochondrial dysfunction, and the disruption of metal homeostasis [63]. The downstream consequences of neuropathological processes contribute to neurodegeneration, with extensive neuronal loss, synaptic changes, and brain neurotoxic events leading to macroscopic atrophy [18,64].

### 3.2. Parkinson’s Disease

Initially described by the English surgeon James Parkinson in 1817, Parkinson’s disease is the second most prevalent ND and the most common movement disorder [65]. 

Clinical manifestations of PD include four cardinal motor symptoms: bradykinesia, resting tremor, rigidity, and postural instability [66,67]. Patients with PD may also experience numerous non-motor symptoms, such as autonomic deficiency, cognitive impairment, neuropsychiatric problems (mood, cognition, behavior, or thought alterations), and both sensory (especially altered sense of smell) and sleep disorders [7,67,68,69]. Non-motor symptoms are common in the PD early stages (pre-motor/prodromal phase) and frequently precede the onset of motor symptoms [68,69]. Motor dysfunction worsens with the disease progression and is managed with symptomatic treatments [69]. However, long-term therapy is associated with the gradual loss of efficacy and the emergence of adverse effects such as motor fluctuations, dyskinesia, and psychosis [69,70]. Late-stage PD is characterized by treatment-resistant motor and non-motor symptoms that substantially contribute to the patient’s disability [69]. The median age of the onset of PD is 60 years, and the mean duration from diagnosis to death is 15 years [67].

Parkinson’s disease is mostly sporadic, resulting from a complex interplay between genetic susceptibility and environmental factors [66]. However, approximately 5–10% of PD cases are caused by familial genetic mutations [71,72] that usually result in early onset PD [73]. Mutations in *SNCA*, *LRRK2* and *VPS35* genes were associated with autosomal dominant PD, while mutations in *PINK1*, *PARK7/DJ-1*, *PARK2/PARKIN*, *PLA2G6*, *ATP13A2*, and *FBXO7* cause autosomal recessive PD and/or parkinsonism [74]. Despite being extensively studied, the gun trigger that causes PD remains unknown. An appraisal of the literature points towards a complex multifactorial etiology, in which a multiplicity of pathological *stimuli* contributes to the neurodegenerative cascade. So far, the main causes include impaired calcium homeostasis, iron overload, inflammation, protein aggregation, and defective metabolism [75]. In addition, several studies showed that oxidative stress can cause neuronal death and mitochondrial dysfunction [76].

**Figure 3:**
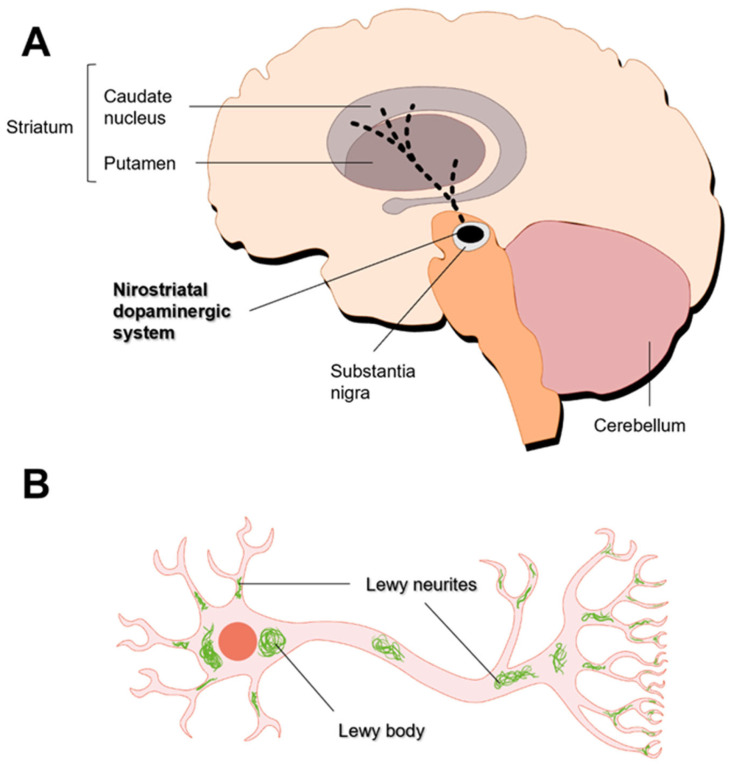
Major hallmarks of Parkinson’s disease. (**A**) Degeneration of nigrostriatal dopaminergic neurons. (**B**) Formation of Lewy inclusions. Adapted from [80,81].

The motor dysfunction observed in PD is linked to the loss of dopaminergic neurons in specific areas of the *substantia nigra pars compacta* (SNpc) region of the midbrain, which contributes to severe dopamine (DA) deficiency in the putamen and the caudate nucleus [77]. The cell bodies of nigrostriatal neurons are in the SNpc and their axon terminals are projected to the dorsal striatum (i.e., putamen and caudate nucleus) (Figure 3A) [5,72]. Dopamine synthesized in this brain region is directed to the striatum and frontal cortex, allowing for the control of the musculoskeletal system and movement. Therefore, the degeneration of dopaminergic neurons leads to a decrease in DA levels. Symptoms of PD only develop after the loss of 50–60% of nigral neurons and the depletion of 70–85% of DA levels [67,78]. Although the neuropathology of PD is primarily characterized by dopaminergic neuron loss, neurodegeneration also extends to other neurotransmitter systems [68]. Indeed, cholinergic (nucleus basalis of Meynert, dorsal nucleus of vagus), serotonergic (raphe), and noradrenergic (locus coeruleus) neurons are also affected [79].

One pathological hallmark of PD is the formation of Lewy bodies, which are lamellated and fibrillated aggregates that include α-synuclein (αSyn) and ubiquitin [82]. The accumulation of αSyn results in the death of dopaminergic neurons [83]. In dopaminergic neurons, αSyn regulates the synthesis, storage, and release of DA [84]. αSyn is prone to form oligomeric and fibrillar bodies in the cytosol or associate to the cellular membrane [85]. The formation of αSyn inclusions begins in the lower brainstem nuclei [86,87], spreads through the pons to the midbrain and basal forebrain and reaches the neocortex [86]. These inclusions may accumulate in neuronal perikarya (Lewy bodies) and neuronal processes (Lewy neurites) (Figure 3B) [88,89]. The presence of these aggregates is associated with the accumulation of synaptic vesicles, decreased DA release, the impairment of degradation pathways, and increased oxidative stress [84].

## 4. Pharmacotherapy of Alzheimer’s and Parkinson’s Diseases

Neurodegeneration is a complex process resulting from multiple defects [90]. The most obvious pathological features of AD and PD include the selective loss of neuronal populations with a consequent decrease in neurotransmitter levels, and the formation of protein aggregates [66,91]. These observations led to the identification of the primary brain enzymatic targets (e.g., cholinesterases (ChEs), monoamine oxidases (MAOs), and catechol-*O*-methyltransferase (COMT)) [90], which are related to the regulation of neurotransmitter levels, and to the subsequent development of the currently available therapeutic agents [92,93].

### 4.1. Targeting Neurotransmitter Depletion in Alzheimer’s Disease

Cholinergic neurons are widely distributed in both the central and the peripheral nervous systems [48]. Although they represent less than 1% of neurons in the nervous system, almost every brain region and peripheral target receives cholinergic innervation [94]. Cholinergic neurons in the basal forebrain contain extensive cortical projections that are involved in the modulation of other neurotransmitter systems [95]. 

Studies focusing on the cholinergic system have received particular attention since the decline of cholinergic function was linked to age-related learning impairments and memory loss in AD [96]. The damage or the presence of abnormalities in cholinergic pathways, especially in the basal forebrain neurons, was correlated with the level of cognitive decline in late-stage AD patients [97]. Together with the loss of cholinergic markers, such as choline acetyltransferase (ChAT) and AChE, these observations led to the formulation of the “cholinergic hypothesis” [98], which states that the dysfunction of the cholinergic system contributes to the cognitive deficits in AD [99].

Acetylcholine (ACh) was the first neurotransmitter to be identified [100,101] and is widely distributed in the nervous system, playing important functional roles in attention, memory, learning, stress response, wakefulness and sleep, and sensory information [102]. The hippocampal and cortical levels of ACh in the brain of AD patients are decreased by approximately 90% [103]. It also has a very important role in the structural and functional remodeling of cortical circuits by establishing synaptic contacts in the networks of cells [104,105].

**Figure 4:**
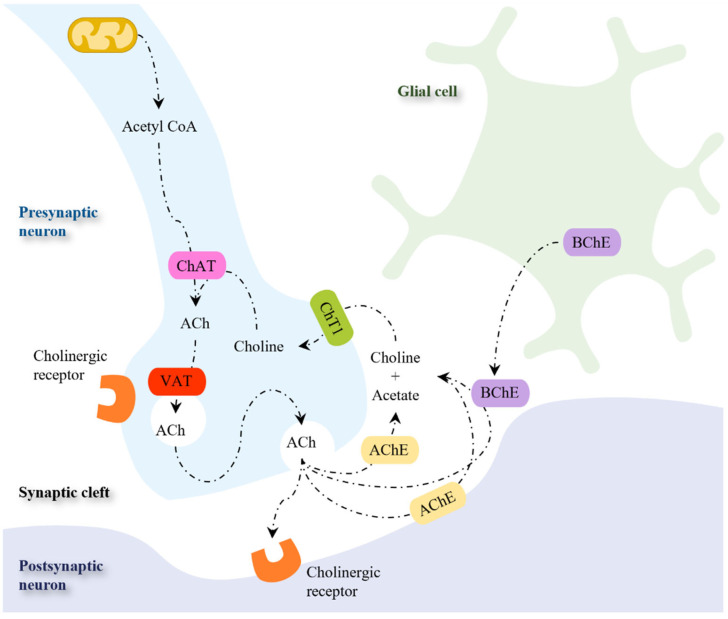
Enzymes and transporters involved in the synthesis, storage, and metabolism of acetylcholine. Abbreviations: Ach, acetylcholine; AchE, acetylcholinesterase; BchE, butyrylcholinesterase; ChT1, high-affinity choline transporter; VAT, vesicular acetylcholine transporter. Adapted from [110,113].

The synthesis of ACh is catalyzed by ChAT in the cytosol of presynaptic cholinergic neurons in a single-step reaction, in which choline and acetyl-coenzyme A (acetyl-CoA) are used as substrates (Figure 4) [106]. While acetyl-CoA is synthesized by mitochondria, choline is taken up from the extracellular space since it is not synthesized in neurons [107]. The rate-limiting step for the synthesis of ACh is the uptake of choline by the Na^+^-dependent, high-affinity choline transporter (ChT1) [108]. The neurotransmitter ACh is then accumulated in synaptic vesicles by the vesicular acetylcholine transporter (VAT). This transporter uses an electrochemical gradient generated by a proton adenosine triphosphate (ATP)ase to perform the uptake of one ACh molecule in exchange for two protons [107,108,109]. During neurotransmission, ACh is released from the presynaptic neuron into the synaptic cleft, where it binds to cholinergic receptors (muscarinic or nicotinic) in the postsynaptic and presynaptic membranes [106,110]. 

The action of ACh may persist for a long time due to the chemical stability of the neurotransmitter [111]. Therefore, the rapid hydrolysis of ACh by cholinesterases is a process to prevent cholinergic overactivation [112]. Two ChEs are present in mammals and can metabolize Ach: acetylcholinesterase (AchE) and butyrylcholinesterase (BchE) [98]. While the Ch obtained from Ach inactivation is taken up by a pre-synaptic neuron via ChT1 [98,106,111], acetic acid is further decomposed [111].

**Figure 5:**
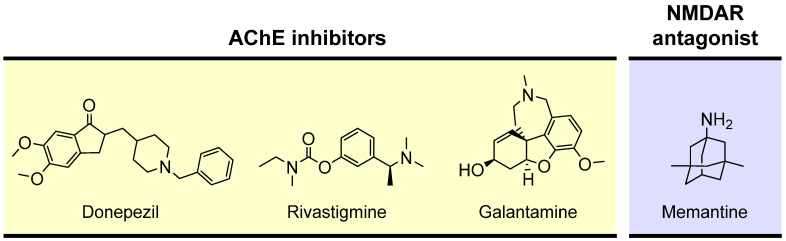
Drugs in clinical use for AD treatment.

Although different approaches have been investigated to improve cholinergic neurotransmission by modulating ACh release [114,115], cholinesterase inhibitors are the only pharmacological strategy approved so far. Cholinesterase inhibitors enhance cholinergic neurotransmission through the inhibition of ChEs, thereby decreasing the breakdown of ACh and increasing its levels at the synaptic cleft. In healthy human brains, AChE represents 80% of the ChEs activity, while BChE plays a supporting role [116]. Over the progression of AD, AChE levels decrease as much as 85% in specific brain areas [95], while BChE levels increase, possibly as a result of glial cell proliferation [113]. As a result, the ratio of BChE to AChE in the cortical areas of the brain shifts from 0.2 to as much as 11 [95]. In AD, the elevated BChE levels are a compensatory mechanism for ACh metabolism [113]. Therefore, the inhibition of both AChE and BChE is considered desirable for the effective management of AD [90,117]. AChE inhibitors are used to treat cognitive and behavioral symptoms of AD patients. Currently available AChE inhibitors used in AD therapy include donepezil, rivastigmine, and galantamine (Figure 5) [118].

AChE inhibitors can be prescribed with memantine (Figure 5), an uncompetitive and low-affinity N-methyl-D-aspartate (NMDA) receptor (NMDAR) antagonist approved for the treatment of moderately severe to severe AD [119,120,121]. The NMDAR is an ionotropic receptor of glutamate, the main neurotransmitter in CNS [115]. The activation of NMDAR generates a long-lasting influx of Ca^2+^ into neurons, which isthought to be involved in the cellular processes underlying learning and memory [122,123]. In AD, an increase in extracellular glutamate is observed, leading to the excessive activation of NMDAR with consequent intracellular accumulation of Ca^2+^ and neuronal death [124]. By blocking excessive NMDAR activation, memantine antagonizes glutamate-mediated excitotoxicity and prevents neuronal cell death [125]. 

### 4.2. Targeting Neurotransmitter Depletion in Parkinson’s Disease

Dopamine is a catecholamine neurotransmitter present in the CNS and in some peripheral areas [126]. In the brain, DA transmission is associated with the control of fine motor movements and with cognitive functions that include learning, reward, attention, and decision-making [126,127]. The DA released in the nigrostriatal pathway is linked to the performance of voluntary movements, as well as the selection and initiation of suitable motor actions [128]. 

**Figure 6:**
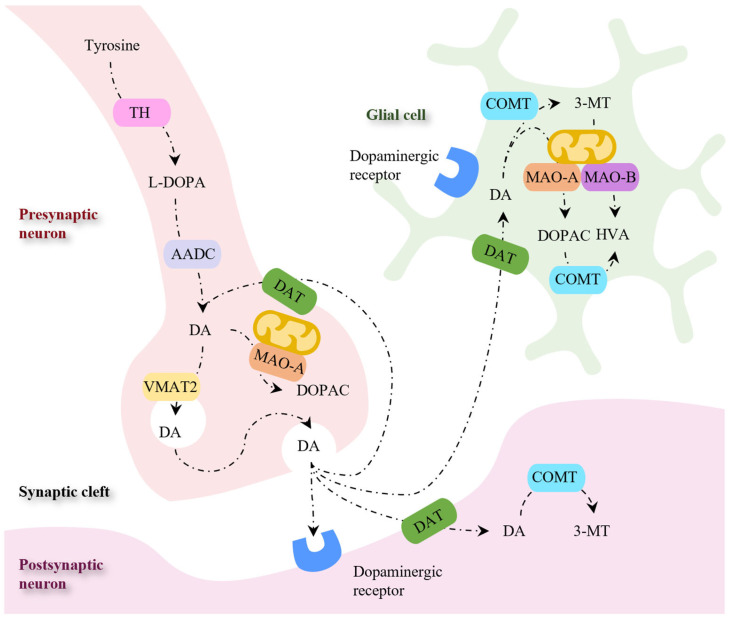
Enzymes and transporters involved in the synthesis, storage, and metabolism of dopamine. Abbreviations: 3-MT, 3-methoxytyramine; AADC, aromatic amino acid decarboxylase; COMT, catechol-O-methyltransferase; DA, dopamine; DAT, dopamine transporter; DOPAC, 3,4-Dihydroxyphenylacetic acid; HVA, homovanillic acid; L-DOPA, L-3,4-dihydroxyphenylalanine; MAO-A, monoamine oxidase A; MAO-B, monoamine oxidase B; TH, tyrosine hydroxylase; VMAT2, vesicular monoamine transporter. Adapted from [130,131].

In the cytoplasm of the presynaptic dopaminergic neuron, DA biosynthesis occurs in two steps (Figure 6). The first step involves the hydroxylation of tyrosine into L-3,4-dihydroxyphenylalanine (L-DOPA) by tyrosine hydroxylase (TH), which is followed by a decarboxylation reaction catalyzed by aromatic amino acid decarboxylase (AADC) to afford DA [81,129]. The neurotransmitter is then transported into synaptic vesicles by the vesicular monoamine transporter (VMAT2) or metabolized by intraneuronal monoamine oxidase A (MAO-A) [130]. Following the release into the synaptic cleft, DA binds to the dopaminergic receptors present in the postsynaptic neuron [129]. The transport of the released DA into the presynaptic neuron occurs via a DA transporter (DAT) and is followed by DA recycling into the synaptic vesicles or by DA deamination by MAO-A. Alternatively, the DA transported into non-dopaminergic post-synaptic neurons and glial cells is metabolized by MAO-B and COMT [130].

The loss of nigrostriatal dopaminergic neurons is associated with the development of the motor symptoms of PD, namely the difficulty in initiating and terminating movements, gait disturbance, and muscular rigidity [128]. Therefore, to reduce the severity of motor handicaps, most PD therapies are based on enhancing the dopaminergic signaling [92,132].

**Figure 7:**
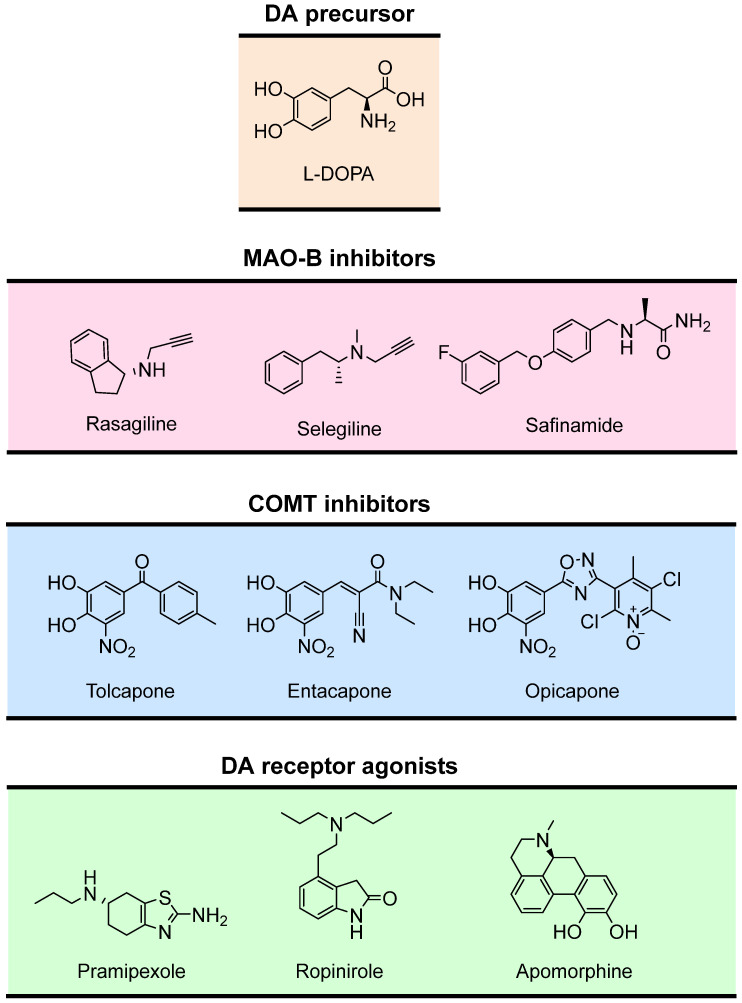
Drugs in clinical use for PD treatment.

The use of the DA precursor L-DOPA (Figure 7) remains the gold-standard treatment for PD [133]. Unlike DA, L-DOPA can cross the blood–brain barrier (BBB) and increase DA synthesis in the brain [129]. To prevent the peripheral metabolic activation to DA, L-DOPA is commonly administered with decarboxylase inhibitors (e.g., carbidopa and benserazide) [134]. Despite the efficacy of L-DOPA in ameliorating motor symptoms, its long-term use is associated with the progressive loss of efficacy together with motor fluctuations and dyskinesia [70,135]. 

In addition to L-DOPA, other therapeutic approaches involve the use of selective MAO-B inhibitors (rasagiline, selegiline, safinamide), COMT inhibitors (entacapone, tolcapone and opicapone) [136,137], or the agonists of postsynaptic DA receptors (pramipexole, ropinirole, apomorphine) (Figure 7) [138,139]. 

Due to their pivotal role in neurotransmitter catabolism and their affinity for specific neurotransmitters, MAOs are considered attractive drug targets in the treatment of depression and NDs [140,141]. In particular, MAO-B is the main isozyme involved in DA metabolism in the aged Parkinsonian brain [142]. While MAO-A activity is maintained with aging, the activity and expression of MAO-B in the human brain increase approximately 4-fold in most brain areas such as the basal ganglia [142,143], possibly as a result of glial cell proliferation and the concomitant loss of neuronal cells [144]. The amplified MAO-B activity leads to nigrostriatal DA depletion and to a higher production of H_2_O_2_ and toxic aldehydes, which contribute to increased oxidative stress and neuronal degeneration [142,143].

MAO-B inhibitors (Figure 7) are currently used in PD therapies to prevent DA catabolism and prolong the action of DA in the basal ganglia [145]. The inhibition of MAO-B may also decrease the formation of dopamine-derived oxidative products, thereby delaying the disease progression [146]. Usually, MAO-B inhibitors are prescribed either as monotherapy or in combination with L-DOPA. Their use in monotherapy is more effective at the early stages of PD and may delay the use of L-DOPA [147]. When combined with L-DOPA, MAO-B inhibitors prolong the therapeutic effects of L-DOPA, decrease the dose of L-DOPA required to control the symptoms, and reduce the occurrence of L-DOPA-associated side effects [142,147]. 

COMT has received considerable attention due to its involvement in the metabolism of L-DOPA [148]. Given adjunctively with L-DOPA, COMT inhibitors (Figure 7) decrease L-DOPA premature inactivation, prolonging its half-life and improving its delivery to the brain [148,149]. In addition, COMT inhibitors enable a decrease in both the dose and administration frequency of L-DOPA, reducing “off” time (i.e., decreasing periods of time when symptoms are more noticeable and movements are more difficult) and increasing “on” time (i.e., increasing periods when PD patients experience good symptom control), thereby improving and prolonging the clinical response to L-DOPA [149]. 

Inhibitors of peripheral (entacapone, opicapone, Figure 7) and cerebral COMT (tolcapone, Figure 7) were developed and are available for the adjunctive treatment of PD. Peripheral COMT inhibition decreases the systemic decomposition of L-DOPA. Still, COMT inhibition in the CNS has the additional advantage of decreasing the metabolism of both L-DOPA and DA in the brain [130].

### 4.3. Alzheimer’s and Parkinson’s Diseases: Looking for New Targets

Currently, the pharmacotherapy for AD and PD consists of drugs approved by the Food and Drug Administration (FDA) that regulate neurotransmitter levels. Unfortunately, they only provide valuable but modest symptomatic benefits, being unable to modify the course of these diseases [9,150]. These treatments are also accompanied by limitations. For instance, AChE inhibitors offer relatively short-lasting positive effects in AD patients [151] and display cholinomimetic actions on the gastrointestinal tract that result in diarrhea, nausea, and vomiting [152]. The efficacy of PD medicines also decreases over time, and the chronic treatment often culminates in motor complications (e.g., L-DOPA-induced dyskinesia) [153].

The need for beneficial neuroprotective agents has been the driving force for the development of new and innovative therapeutic strategies, preferably with disease-modifying outcomes. For instance, over the last years, efforts have been made to develop new drug candidates able to tackle increased oxidative stress, metal dyshomeostasis (iron, copper), neuroinflammation, and the aggregation of misfolded protein (Table 1). The following subsections will discuss the development of pharmacological agents targeting oxidative stress or the adenosine receptors in NDs as representative examples. 

#### 4.3.1. Oxidative Stress as a Target in Alzheimer’s and Parkinson’s Diseases

Oxidative stress is one of the major contributors to the pathogenic cascade that leads to neurodegeneration in AD and PD [209,210]. Evidence of reactive oxygen species (ROS)-mediated injuries, with increased levels of oxidative markers and damaged cell components, were observed in AD and PD brains [211]. A decline in the pool of endogenous antioxidants and a decrease in the activity of antioxidant enzymes were also reported [25,212]. 

The brain is particularly prone to oxidative stress-induced damage. Although the brain constitutes only ~2% of the total body weight, it is responsible for more than 20% of the body’s oxygen consumption, with a significant amount of oxygen being converted into ROS [210,213,214]. Despite this massive oxygen consumption, the brain presents a lower content of endogenous antioxidants (e.g., glutathione and catalase) in comparison to other tissues, thus being more sensitive to cellular redox dyshomeostasis [213,215]. In addition, redox-active metals (e.g., iron and copper) accumulate in specific brain regions and catalyze the formation of ROS [122,210]. Finally, the high levels of polyunsaturated fatty acids in the brain increase the susceptibility to lipid peroxidation and subsequent formation of toxic compounds [210,213,216]. 

**Figure 8:**
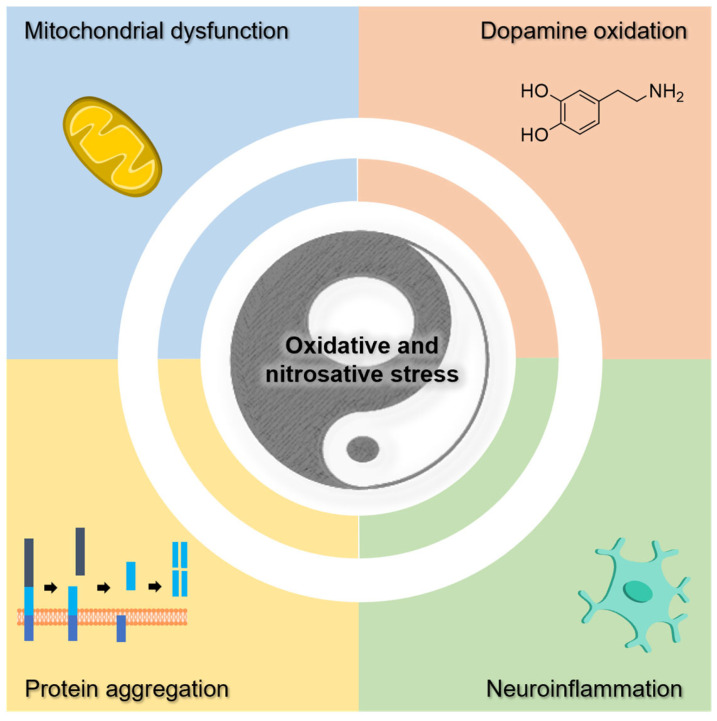
Pathological events of neurodegenerative diseases associated with increased oxidative stress.

The increased oxidative stress in NDs is strictly connected to other pathological events, namely mitochondrial dysfunction, dopamine oxidation, neuroinflammation, and the accumulation of protein aggregates (e.g., Aβ and α-syn) (Figure 8) [217].

**Figure 9:**
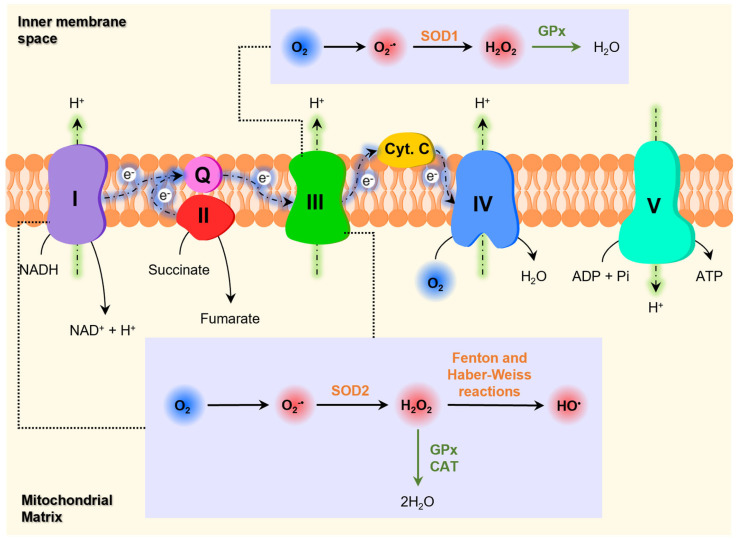
Formation of ROS in mitochondria. Abbreviations: ADP, adenosine diphosphate; ATP, adenosine triphosphate; CAT, catalase; Cyt C, Cytochrome C; GPx, Glutathione peroxidase; I, complex I; II, complex II; III, complex III; IV, complex IV; NADH, Nicotinamide adenine dinucleotide; Q, coenzyme Q10; SOD1, superoxide dismutase 1; SOD2, superoxide dismutase 2; V, complex V (ATP synthase). Adapted from [119,220].

Although ROS are generated in several cellular compartments, mitochondria is one of the main sources of the overproduction of ROS [123]. The formation of ROS in mitochondria occurs primarily at the ETC present in the inner mitochondrial membrane (Figure 9) [124,210]. The mitochondrial ETC consists of a series of membrane-bound complexes (complexes I, II, III and IV) [119], which generate a proton gradient across the inner mitochondrial membrane through electron transfers, leading to the production of ATP by ATP synthase (complex V) [210]. Metabolic intermediates formed during the Krebs cycle are used for oxidative phosphorylation [120]. During the ETC, a small proportion of electrons occasionally leak and directly reduce O_2_ to $O_{2}^{•-}$ [120,121], which is, in turn, converted into other ROS such as H_2_O_2_ and HO^•^ [123]. The formation of $O_{2}^{•-}$ occurs mainly in complexes I and III [121,218]. Enzymes from the Krebs cycle (e.g., α-ketoglutarate dehydrogenase, pyruvate dehydrogenase, and aconitase) may also generate ROS [218,219].

Mitochondrial ETC is one of the primary targets of the harmful effects inflicted by high levels of ROS [119,221]. The oxidative damage at this level leads to the inhibition of ATP synthesis and the increased production of ROS in a vicious and detrimental cycle, contributing to cell dysfunction and cell death [124,221,222]. Mitochondria contain other components susceptible to oxidative damage, namely several iron-sulfur centers, proteins and unsaturated fatty acids in the inner membrane, and mitochondrial DNA (mtDNA), all of which are important for proper mitochondrial function [124]. Considering that mtDNA encodes some of the subunits of the complexes that constitute the ETC, the oxidative damage of mtDNA leads to the defective production of these proteins and subsequent mitochondrial dysfunction [221]. 

Since neurons have limited glycolytic capacity, they are particularly dependent on mitochondrial oxidative phosphorylation to meet their high energy requirements [222,223,224]. In addition to ATP synthesis, mitochondria are involved in other crucial cellular functions such as the synthesis of amino acids and steroids, β-oxidation of fatty acids, Ca^2+^ homeostasis, and the regulation of apoptotic cell death [225]. Therefore, improper mitochondrial function compromises neuronal survival and contributes to neurodegeneration [219,225]. 

**Figure 10:**
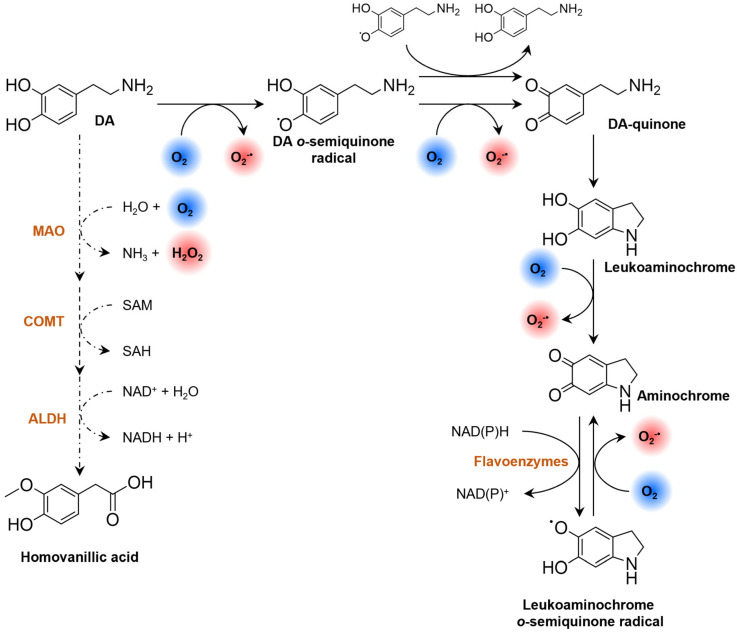
Generation of ROS during enzymatic (dashed line) and non-enzymatic (plain line) DA decomposition. Abbreviations: ALDH, aldehyde dehydrogenase; COMT, catechol O-methyltransferase; DA, dopamine; DA-quinone, dopamine quinone; MAO, monoamine oxidase, NAD(P)H, nicotinamide adenine dinucleotide (phosphate). Adapted from [229].

In PD, DA oxidation is associated with a selective vulnerability of dopaminergic neurons to oxidative stress [220]. Despite the essential role of DA in neurotransmission, DA contains a catechol group that may participate in the generation of ROS and metal chelation [226]. Dopamine is normally stored in monoaminergic vesicles under a low pH environment that prevents its oxidation [227]. However, DA may undergo enzymatic and non-enzymatic decomposition in the cytosol, which is accompanied by the formation of ROS (Figure 10) [228].

In the presence of O_2_, DA generates $O_{2}^{•-}$ and electron-deficient DA semiquinones and DA quinones (Figure 10) [228,230,231]. The reaction rate of DA semiquinone formation is slow, but it is accelerated by redox-active transition metals [215]. The spontaneous cyclization of DA quinone yields leucoaminochrome whose further autoxidation forms aminochrome and $O_{2}^{•-}$ [230]. Aminochrome participates in redox-cycling reactions that result in the formation of $O_{2}^{•-}$ and in the depletion of cellular nicotinamide adenine dinucleotide (NADH) and nicotinamide adenine dinucleotide phosphate (NADPH) [232]. Dopamine quinone and aminochrome also form adducts, with cellular nucleophiles modifying their function [233,234]. These include DNA, biothiols (e.g., glutathione), α-synuclein and proteins involved in ATP synthesis (complexes I, III and V of the ETC), proteasomal degradation (parkin), microtubule stabilization (α- and β-tubulin) and axonal transport (actin) [229]. Therefore, the formation of these adducts will contribute to mitochondrial dysfunction, the impairment of the axonal transport, the inhibition of the proteasomal system, the disruption of cytoskeleton architecture, and the formation of α-synuclein aggregates in PD [229]. Aminochrome also polymerizes into neuromelanin, a brain pigment that contributes to neurodegeneration by triggering neuroinflammatory processes [210].

The oxidative deamination of DA by MAOs uses O_2_, and generates H_2_O_2_ and ammonia as by-products (Figure 10) [235]. Due to the increased expression with age in neuronal tissue [102,236], MAO-B becomes the predominant isoform involved in DA metabolism [210]. Monoamine oxidase B is mainly found in glial cells [105,237], but the H_2_O_2_ produced during DA deamination can permeate cell membranes and induce toxic effects in the neighboring neurons [210]. In fact, compared with astrocytes, neurons are more vulnerable to H_2_O_2_ due to the lower content in antioxidants involved in its detoxification (e.g., GPx and glutathione) [237]. The H_2_O_2_ generated from MAO-B activity in astrocytes is also associated with increased amyloid plaque deposition [111].

Neuroinflammation represents a set of inflammatory processes occurring in the central nervous system that involve the action of glial cells in CNS (microglia, oligodendrocytes, astrocytes), non-glial resident myeloid cells (macrophages and dendritic cells) and peripheral leukocytes [238,239]. Neuroinflammation plays an important role in the progression of NDs [228]. For instance, in AD, microglia are activated by the presence of Aβ and co-localize with the plaques [240]. However, instead of efficiently removing the Aβ deposits, microglia release pro-inflammatory mediators that lead to neuronal damage [241]. In PD, extracellular αSYN aggregates can also interact with and activate surrounding glial cells to trigger a deleterious pro-inflammatory response [242]. In NDs, the expression of NADPH oxidases (NOXs) in activated microglia and reactive astrocytes is increased, resulting in the excessive formation of $O_{2}^{•-}$ [228,243]. The activation of RS-producing enzymes in glial cells is associated with neurotoxic effects, which arise not only from the direct oxidative damage in neurons, but also from the intracellular redox signaling that exacerbates the pro-inflammatory response [243,244]. 

##### Targeting Oxidative Stress with Mitochondria-Targeted Antioxidants

Considering the involvement of oxidative stress in the pathophysiology of NDs, the rationale for using exogenous antioxidants to prevent delay or remove the oxidative damage is evident [214,245]. In fact, several exogenous antioxidants showed promising results in animal and cellular models [213,214]. However, the results obtained in clinical trials were inconclusive, negative, or showed little benefit in NDs [246]. Numerous factors contribute to the discrepancy between pre-clinical and clinical results. In addition to the aspects associated with the design of clinical trials (e.g., posology, the duration of treatment, age, and the disease stage of the patients), most known dietary antioxidants display poor bioavailability and are unable to cross the BBB, affecting their delivery into the brain [213,214,246,247].

A common strategy used to overcome these pharmacokinetic limitations is the introduction of minor structural modifications on the antioxidant scaffold. The resulting derivatives may improve the targeting and drug-like properties while preserving or enhancing the antioxidant profile of the parent compounds [248,249]. 

Aside from the pharmacokinetic constraints, the lack of the clinical efficacy of antioxidants may also result from the uniform distribution of antioxidants across all tissues and organs following administration, with only a small fraction being taken up by mitochondria [246,250], the main source and the target of ROS. Therefore, the development of antioxidants that selectively accumulate within mitochondria and tackle oxidative damage is of particular interest [251]. Compounds lacking mitochondriotropism but with relevant biological activities towards mitochondrial targets usually need to be directed to mitochondria [252]. In this sense, several approaches were developed to deliver antioxidants and other bioactive molecules to mitochondria, but one of the most widely used is their conjugation with lipophilic cations such as triphenylphosphonium (TPP^+^) [251,253]. 

**Figure 11:**
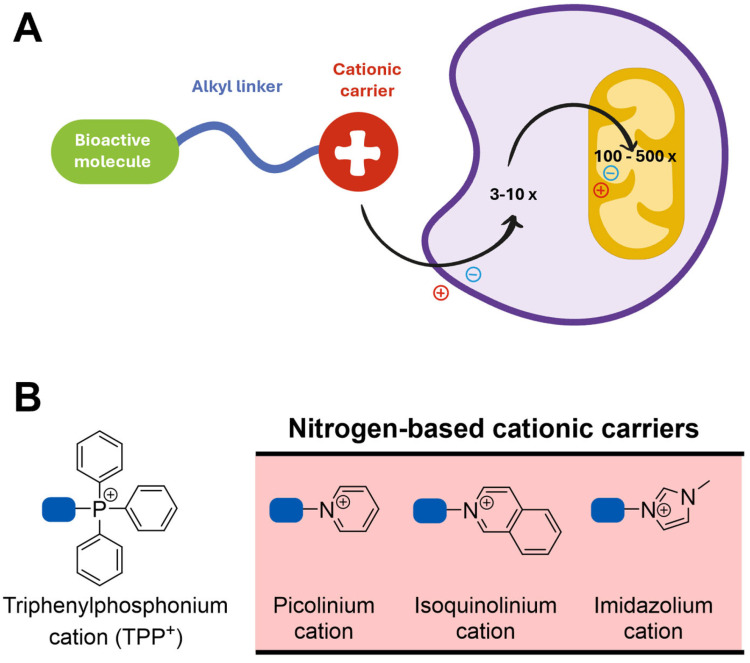
(**A**) Mitochondrial uptake of lipophilic cations. (**B**) Representative examples of lipophilic cations to target bioactive molecules to mitochondria. Adapted from [255,257].

Lipophilic TPP^+^ cations can diffuse across phospholipid bilayers because their positive charge is surrounded and dispersed over a large hydrophobic surface area, which decreases the activation energy for membrane permeation [254,255,256]. In response to the plasma and mitochondrial membrane potentials (${\Delta \Psi }_{plasma}\;and\;{\Delta \Psi }_{mitochondria}$, respectively), these compounds accumulate within the mitochondrial matrix against the concentration gradient [254] (Figure 11A). Then, TPP^+^ conjugates are taken up from the intracellular space to the mitochondrial matrix in response to the ${\Delta \Psi }_{mitochondria}$ (−140 to −160 mV), leading to 100- to 500-fold accumulation within the mitochondrial matrix [220,256]. 

The increased accumulation of lipophilic TPP^+^ conjugates enhances the compounds’ potency and decreases the external dose required, limiting the extramitochondrial metabolism that results in inactivation, excretion, or toxicity [124,258]. However, the extensive accumulation of these compounds within the mitochondrial matrix can disrupt membrane integrity, thereby compromising cellular respiration and ATP production [259,260]. 

Following oral or intravenous administration, lipophilic TPP^+^ conjugates are rapidly taken up by the organs most affected by mitochondrial dysfunction (e.g., liver, heart, and brain) [254,261]. Therefore, targeting antioxidants in mitochondria stands out as a promising strategy in the discovery of new therapies for oxidative stress-related disorders. 

Over the last decade, TPP^+^ cations have been conjugated with dietary antioxidants such as hydroxybenzoic [262] and hydroxycinnamic acids [263]. These compounds displayed remarkable antioxidant properties and were able to protect neuroblastoma cells against the oxidative damage induced by 6-hydroxydopamine or H_2_O_2_ [264]. Moreover, in studies performed in skin fibroblasts from male sporadic PD (sPD) patients, the caffeic acid-based TPP^+^ conjugate AntiOXCIN4 restored mitochondrial membrane potential and mitochondrial fission, decreased autophagic flux, and enhanced cellular responses to stress by improving the cellular redox state and decreasing ROS levels [265]. To circumvent the drawbacks associated with the use of the TPP^+^ cation, its replacement with nitrogen-based cationic carriers (e.g., isoquinolinium, imidazolium, and picolínium) was recently performed (Figure 11B) [266]. This chemical modification resulted in decreased cytotoxicity while maintaining the compounds’ antioxidant properties and their ability to accumulate within mitochondria [266].

#### 4.3.2. Adenosine Receptors as a Target in Alzheimer’s and Parkinson’s Diseases

**Figure 12:**
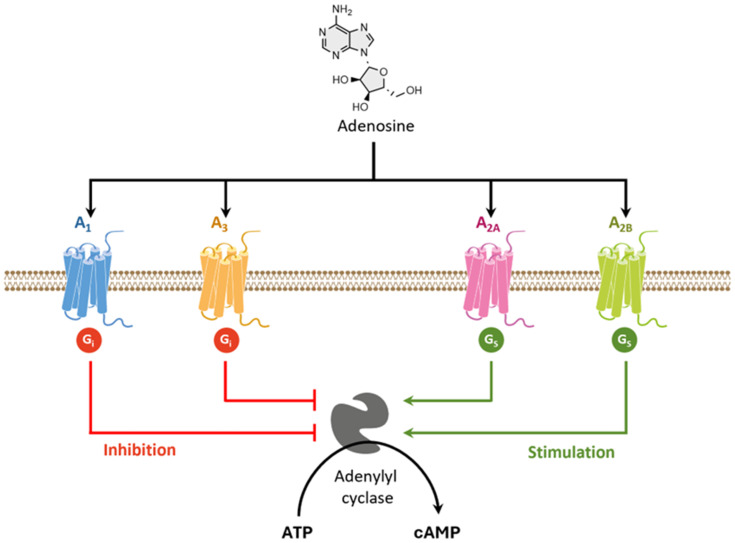
Schematic representation of G protein-coupled adenosine receptors.

Adenosine is a purine nucleoside that may act as a neurotransmitter as neuromodulator in the CNS [267]. It is involved in several physiological and pathophysiological processes in the brain, including motor function, sleep/wake cycle, learning and memory, pain, and astrocytic activity [268]. To perform its physiological roles, adenosine binds to four distinct G-coupled protein adenosine receptors (ARs), designated as A_1_, A_2A_, A_2B_ and A_3_. Adenosine receptors (ARs) represent a group of glycoproteins containing seven transmembrane domains and are coupled to different G proteins [269] (Figure 12). While adenosine A_1_ and A_3_ receptors are coupled to inhibitory G proteins, A_2A_ and A_2B_ ARs are coupled to stimulatory G proteins. The A_2A_ and A_2B_ ARs preferably interact with members of the G_s_ family of G proteins, stimulating adenylyl cyclase to produce cyclic AMP (cAMP) and leading to the activation of a series of downstream signaling pathways. In contrast, A_1_ and A_3_ ARs inhibit adenylyl cyclase activity by interacting with G_i_ proteins (Figure 12) [270]. 

ARs are widely distributed in the human body and participate in a broad range of physiological and pathophysiological processes [198]. While A_1_Rs and A_2_Rs can be predominantly found in specific parts of the CNS, A_2B_Rs and A_3_Rs are mainly located in peripheral tissues [271].

In the CNS, A_1_Rs are widely distributed in neocortical and limbic systems and are linked to cognitive functions [198,272,273]. A_2A_Rs are highly expressed in striatal areas [198,272] and participate in the regulation of motor behavior and the management of dopamine-mediated responses [199]. A_2A_Rs co-localize with dopamine D_2_ receptors (D_2_Rs) on GABAergic striatopallidal output neurons, where they form heteromer complexes [274]. These receptors within the heteromeric complex exert opposite effects on motor behavior, in which A_2A_ AR agonism induces antagonistic effects on D_2_Rs. For instance, the stimulation of dopamine D_2_Rs enhances motor activity, while A_2A_ ARs decrease this effect by decreasing the affinity and response of D_2_Rs to their ligands [273,275].

Excessive A_2A_ AR function has been linked to neuronal damage [276], and increased A_2A_ AR expression is a characteristic feature of PD progression [277]. The cellular mechanisms responsible for A_2A_ AR-mediated neurodegeneration remain elusive. However, evidence suggests that the activation of A_2A_ ARs leads to increased glutamate release, increased Ca^2+^ entry, and enhanced long-term potentiation, all of which may culminate in excitotoxic damage [275]. The localization of A_2A_ ARs at the basal ganglia, coupled with their pathophysiological role in PD, makes these receptors attractive drug targets to treat this disease [275]. A_2A_ AR antagonism decreases motor impairment by enhancing dopamine D_2_R-mediated signaling. Moreover, A_2A_ AR antagonism modulates cholinergic, glutamatergic, and GABAergic functions in the CNS [273]. The blockade of A_2A_ AR signaling with selective A_2A_ AR receptor antagonists was shown to be beneficial, not only by enhancing the therapeutic effects of L-DOPA, but also by reducing dyskinesia from long-term L-DOPA treatment [274].

Recent studies have also disclosed a close association between A_2A_ ARs and cognitive impairment in AD. For instance, abnormally high levels of A_2A_ AR were detected in the hippocampus and in the cortex of AD patients [266,278] and in APP/PS1 transgenic AD mice [279]. Remarkably, the activation of A_2A_ ARs with agonists and optogenetic agents led to severe impairments in spatial discrimination in wild-type mice [280]. The involvement of A_2A_ ARs in in hippocampal-dependent spatial reference memory was also shown in A_2A_ AR knock-out studies in an Aβ_1-42_-based mice model of AD [281]. The memory deficits in APP/PS1 mice were reverted by the blockade of A_2A_ ARs with a selective antagonist or by downregulation driven with shRNA interference [279]. Finally, recently, it was shown that the improvement of spatial memory deficits by A_2A_ AR antagonists in APP/PS1 mice results from the promotion of the synaptic plasticity of adult-born granule cells [282]. Thus, the blockade of A_2A_ AR activation with selective antagonists can be of great therapeutic benefit to AD patients.

##### A2A Adenosine Receptor Antagonists

**Figure 13:**
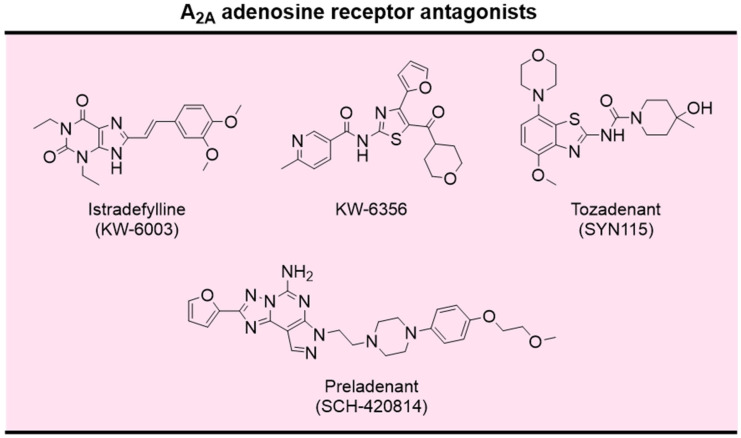
Chemical structures of selective A_2A_ AR antagonists evaluated in clinical trials.

The knowledge acquired over the last decades concerning the involvement of adenosine in motor functions, mainly through the modulation of A_2A_ AR, makes A_2A_ AR antagonists promising non-dopaminergic agents for the treatment of PD motor symptoms. Over the last decades, the development of potent and selective ligands for ARs has been a dynamic area. Excellent reviews were recently published on this topic [283,284]. A small number of selective A_2A_ AR antagonists reached advanced clinical trials for the treatment of motor symptoms in PD, namely the xanthine derivative istradefylline (KW-6002) and the non-xanthine derivatives Tozadenant (SYN115), Preladenant, and KW-6356 (Figure 13) [272,277].

Istradefylline was approved for the adjunctive treatment of PD in Japan in 2013 and by the FDA in 2019, being the first non-dopaminergic drug approved by the FDA for PD in the last two decades [285]. Preladenant and Tozadenant underwent clinical evaluation for the treatment of PD (Preladenant: NCT00406029, NCT01227265; Tozadenant: NCT02453386, NCT03051607) [286]. Unfortunately, the clinical evaluation for both drug candidates was discontinued due to the lack of efficacy (Preladenant) or safety (Tozadenant) in phase 3 clinical trials [287].

KW-6356 is a new, selective, nonxanthine A_2A_ receptor antagonist/inverse agonist. Compared to istradefylline, KW-6356 exhibits approximately 100-times higher affinity for the human A_2A_ receptor and a prolonged drug residence time [288]. In a phase 2b clinical study in patients with PD, KW-6356 was safe and effective in the adjunctive treatment with L-DOPA (NCT03703570) [289]. Moreover, in a phase 2a clinical trial, KW-6356 monotherapy was well tolerated and more effective than placebo in patients with early, untreated PD (NCT02939391) [277].

## 5. Conclusions

The discovery of new drugs for NDs remains an enormous unmet medical need [290]. The available treatments for AD and PD provide valuable symptomatic relief, but only reduce the symptoms for a short period before the cognitive or motor functions continue to deteriorate [291]. Given the lack of therapeutic efficacy in current treatments, the use of single-target drugs may be insufficient to address the multiple pathological aspects of NDs [292]. The treatment of AD and PD may thus require the manipulation of several targets to restore physiological balance, thereby attaining significant therapeutic efficacy [293].

Traditionally, the “one-drug, one-target” paradigm is the mainstay drug discovery concept in the pharmaceutical industry [91]. This paradigm is mainly focused on generating drugs that selectively bind to a single biological target, avoiding potential adverse side effects associated with mistargeting other biological entities [294]. The current therapy for AD and PD management is based on this paradigm. However, current single-target drugs address the diseases’ symptomatology, without halting or modifying the disease progression [294,295]. Therefore, drugs that can simultaneously manipulate multiple targets may provide therapeutic benefits in AD and PD diseases due to their multifactorial nature and complexity [296]. The limited clinical efficacy and the lack of disease-modifying effects of the available drugs shifted the research focus from single-target agents to multitarget-directed drugs [237,247]. The field of the multitarget approach may thus provide innovative therapeutic solutions to feed the pipeline of disease-modifying drugs for AD and PD. The potential of the multitarget approach in discovering new drug candidates for multifactorial diseases such as AD and PD was extensively reviewed in [294,297,298].

Over the last decades, continuous efforts have been made to unveil the pathological mechanisms underlying AD and PD development. Processes such as oxidative stress, metal dyshomeostasis, neuroinflammation, and protein misfolding and aggregation were identified, and new pathological events were discovered daily. As a result, promising drug targets have been emerging, providing excellent opportunities to innovate in developing new drug candidates. Thus, where do we go now? With the growing popularity of the multitarget paradigm, the possibilities are endless, and several paths can be followed. Medicinal chemists can explore multiple combinations of pharmacological effects in the search for disease-modifying drugs with a multitarget mode of action. Another emerging area is the development of new chemical modalities such as protein degraders. Targeted protein degradation (TDP) technologies include proteolysis-targeting chimeras (PROTACs), autophagy-targeting chimeras (AUTACs), and autophagosome-anchoring chimeras (ATACCs). Although the application of TDP technologies for NDs is still in its infancy, recent studies have shown that their use is a strategy worth exploring [299,300]. The combination of TDP technologies with multitarget strategies is also a direction that can be followed.

## Figures and Tables

**Table 1:** Examples of therapeutic targets looked at for drug discovery and the development of AD and PD.

(Patho)physiological Process	Target	
	AD	PD
Metal dyshomeostasis	copper [154]; iron [155]; zinc [156]	Copper [157]; iron [158]
Mitochondria and metabolic functions	MCL1 [159]	ROCK [160], δ-opioid receptor [161]
Mutated/misfolded proteins	Amyloid-β [162]; β-secretase [163]; γ-secretase [164]; GSK3-β [165]; RAGE [166]; Tau [167]	DJ1 [168]; LRRK2 [169]; Pink1 [170]; α-synuclein [171]; CK1δ [172]; CK1δ+GSK3b [173]
Neuroinflammation	NRLP3 inflammasome [174]	Adenosine receptors [175]; cannabinoid receptor 2 [176]; monoacylglycerol lipase [177]; NRLP3 Inflammasome [178]; PPAR [179]; TRPC5 [180]
Neuroprotection	Apoptosis [181]; ferroptosis [182]; sigma 1 and 2 receptors [183]	Apoptosis [184]; autophagy/neuroinflammation [185]; CDNF peptidomimetic [186]; ferroptosis [187]; nurr1 [188]; sigma 1 and 2 receptors [189]
Oxidative stress	Antioxidants [190]; NRF2 signaling pathway [191]	Aldose reductase [192]; NRF2 signaling pathway [193]
Synaptic activity	AChE [110]; α7nAChR [194]; butyrylcholinesterase [195]; NMDA receptor [196]	5-HT2A [197]; adenosine receptors [198,199]; α6AChR [200]; COMT [201]; dopaminergic D1-D4 receptors [202,203,204]; GPR6 [205]; MAO-B [206]; mGlu4 [207]; PDE4 [208]
